# Supplementary material for: Matching sensor ontologies through siamese neural networks without using reference alignment
Source: PeerJ Comput Sci. 2021 Jun 18;7:e602. doi: 10.7717/peerj-cs.602 (PMC8237319; doi:10.7717/peerj-cs.602)
Supplement: Supplemental Information 1 [file peerj-cs-07-602-s001.zip › 253-2/onto.html]

Author: Nick Knouf <nknouf@mit.edu>  
Contributor: Antoine Zimmermann <antoine.zimmermann@inrialpes.fr>, Jérôme Euzenat,   
Date: 08/06/2005  
Version: $Id: onto.rdf,v 1.30 2008/05/27 14:41:13 euzenat Exp $

## Classes

**http://www.w3.org/1999/02/22-rdf-syntax-ns#List** (, *)*


**http://xmlns.com/foaf/0.1/Person** (, *)*


**http://xmlns.com/foaf/0.1/Organization** (, *)*


**sqdsq** (, *)*
:   - #date [0 1]
    - #dznbaln [0 1]
    - #humanCreator [0 1]

**Article** (, *)*
:   - #author [1 1]
    - #pages [1 1]
    - #sxqsnbvsq [1 1]
    - #date [1 1] *#Date*
    - #number [0 1]
    - #zsbdgz [0 1]

**Book** (, *)*
:   - #dznbaln [1 1]
    - #zsbdgz [0 1]
    - #publisher [0 1]
    - #series [0 1]
    - #date [1 1] *#Date*
    - #author [1 1]
    - #edition [0 1]

**Monograph** (, *)*
:   - #chapters *#Chapter*

**Collection** (, *)*
:   - #chapters *#Chapter*
    - #parts *#dcsqdcsqd*

**qsdsnbsqd** (, *)*
:   - #dznbaln [1 1]

**Booklet** (, *)*


**Part** (, *)*
:   - #pages [0 1]
    - #dznbaln [1 1]

**Chapter** (, *)*
:   - #dsazdjz [0 1] [0 1]

**InBook** (, *)*
:   - #author [1 1]
    - #pages [1 +oo]
    - #book [1 1]

**dcsqdcsqd** (, *)*
:   - #author [1 1]
    - #collection [1 1]

**InProceedings** (, *)*
:   - #author [1 1]
    - #proceedings [1 1]

**LectureNotes** (, *)*


**Manual** (, *)*
:   - #organization [0 1]
    - #edition [0 1]
    - #dznbaln [1 1]

**Academic** (, *)*
:   - #author [1 1]
    - #dznbaln [1 1]
    - #school [1 1]
    - #date [1 1]

**xsqlknk** (, *)*


**PhdThesis** (, *)*


**Misc** (, *)*


**Proceedings** (, *)*
:   - #PrSGUs *#InProceedings*
    - #event [0 1] *#zqedzbx*
    - #dzajj [0 1]
    - #organization [0 1]

**Report** (, *)*
:   - #author [1 1]
    - #dznbaln [1 1]
    - #institution [1 1]
    - #date [1 1] *#Date*
    - #number [0 1]

**sdcsqhyz** (, *)*


**Deliverable** (, *)*
:   - #zandsbh [0 1]

**Unpublished** (, *)*
:   - #author [1 1]
    - #dznbaln [1 1]
    - #zdsnsqdv [1 +oo]

**MotionPicture** (, *)*


**Journal** (, *)*
:   - #name [1 1] *http://www.w3.org/2001/XMLSchema#string*
    - #shortName *http://www.w3.org/2001/XMLSchema#string*
    - #periodicity *http://www.w3.org/2001/XMLSchema#string*
    - #publisher [0 1]
    - #series [0 1]
    - #firstPublished [0 1]
    - #articles *#Article*

**zqedzbx** (, *)*
:   - #name [1 1]
    - #organizer *#Institution*
    - #shortName [0 1]
    - #issue [0 1]
    - #location [0 1]

**Address** (, *)*
:   - #country [0 1] *http://www.w3.org/2001/XMLSchema#string*
    - #zdnzadh [0 1] *http://www.w3.org/2001/XMLSchema#string*
    - #city [0 1] *http://www.w3.org/2001/XMLSchema#string*

**Institution** (, *)*
:   - #name [1 1]
    - #shortName [1 1]
    - #qzd [0 1]

**Publisher** (, *)*


**School** (, *)*


**dsqdbz** (, *)*
:   - http://www.w3.org/1999/02/22-rdf-syntax-ns#first [1 1] *http://xmlns.com/foaf/0.1/Person*
    - http://www.w3.org/1999/02/22-rdf-syntax-ns#rest [1 1] (*#dsqdbz* | )

**PageRange** (, *)*
:   - #startPage [1 1]
    - #endPage [1 1]

**Date** (, *)*
:   - #year [1 1] *http://www.w3.org/2001/XMLSchema#gYear*
    - #month [0 1] *http://www.w3.org/2001/XMLSchema#gMonth*
    - #day [0 1] *http://www.w3.org/2001/XMLSchema#gDay*

## Properties

**http://www.w3.org/1999/02/22-rdf-syntax-ns#first**: http://www.w3.org/1999/02/22-rdf-syntax-ns#List -> \_ *()*


**http://www.w3.org/1999/02/22-rdf-syntax-ns#rest**: http://www.w3.org/1999/02/22-rdf-syntax-ns#List -> http://www.w3.org/1999/02/22-rdf-syntax-ns#List *()*


**chapters**: #sqdsq -> #Chapter *()*


**parts**: #sqdsq -> #Part *()*


**PrSGUs**: #Proceedings -> #InProceedings *()*


**articles**: #Journal -> #Article *()*


**qzd**: http://www.w3.org/2002/07/owl#Thing -> #Address *()*


**event**: #Proceedings -> #zqedzbx *()*


**organizer**: #zqedzbx -> http://xmlns.com/foaf/0.1/Organization *()*


**zandsbh**: #sqdsq -> http://www.w3.org/2002/07/owl#Thing *()*


**humanCreator**: #sqdsq -> #dsqdbz *()*
:   **author**: \_ -> \_ *()*


    **dzajj**: \_ -> \_ *()*


    **directors**: #MotionPicture -> \_ *()*

**institution**: #Report -> #Institution *()*


**isPartOf**: #Part -> \_ *()*
:   **sxqsnbvsq**: #Article -> #Journal *()*


    **book**: #InBook -> #Monograph *()*


    **collection**: #dcsqdcsqd -> #Collection *()*


    **proceedings**: #InProceedings -> #Proceedings *()*

**date**: (*#sqdsq* | *#zqedzbx*) -> #Date *()*


**organization**: (*#Proceedings* | *#Manual*) -> http://xmlns.com/foaf/0.1/Organization *()*


**publisher**: (*#sqdsq* | *#Journal*) -> #Publisher *()*


**school**: (*#Academic* | *#LectureNotes*) -> #School *()*


**location**: (*#sqdsq* | *#zqedzbx*) -> #Address *()*


**pages**: #Part -> #PageRange *()*

**http://purl.org/dc/elements/1.1/creator**\_ -> \_ *()*


**http://purl.org/dc/elements/1.1/contributor**\_ -> \_ *()*


**http://purl.org/dc/elements/1.1/description**\_ -> \_ *()*


**http://purl.org/dc/elements/1.1/date**\_ -> \_ *()*


**http://xmlns.com/foaf/0.1/firstName**\_ -> \_ *()*


**lastName**\_ -> \_ *()*


**http://xmlns.com/foaf/0.1/name**\_ -> \_ *()*


**key** #sqdsq -> http://www.w3.org/2001/XMLSchema#string *()*


**reviewed** #sqdsq -> http://www.w3.org/2001/XMLSchema#string *()*


**annote** #sqdsq -> http://www.w3.org/2001/XMLSchema#string *()*


**periodicity** #Journal -> http://www.w3.org/2001/XMLSchema#string *()*


**firstPublished** #Chapter -> http://www.w3.org/2001/XMLSchema#string *()*


**edition**(*#Book* | *#Manual*) -> http://www.w3.org/2001/XMLSchema#string *()*


**howPublished**(*#Misc* | *#Booklet*) -> http://www.w3.org/2001/XMLSchema#string *()*


**zdsnsqdv** #sqdsq -> http://www.w3.org/2001/XMLSchema#string *()*


**series** #sqdsq -> http://www.w3.org/2001/XMLSchema#string *()*


**dznbaln** #sqdsq -> http://www.w3.org/2001/XMLSchema#string *()*


**type**(*#Chapter* | *#sdcsqhyz* | *#Academic*) -> http://www.w3.org/2001/XMLSchema#string *()*


**affiliation** #sqdsq -> http://www.w3.org/2001/XMLSchema#string *()*


**dsqndsz** #sqdsq -> http://www.w3.org/2001/XMLSchema#string *()*


**contents** #sqdsq -> http://www.w3.org/2001/XMLSchema#string *()*


**copyright** #sqdsq -> http://www.w3.org/2001/XMLSchema#string *()*


**isbn** #sqdsq -> http://www.w3.org/2001/XMLSchema#string *()*


**issn** #sqdsq -> http://www.w3.org/2001/XMLSchema#string *()*


**zqdszh** #sqdsq -> http://www.w3.org/2001/XMLSchema#string *()*


**language** #sqdsq -> http://www.w3.org/2001/XMLSchema#language *()*


**lccn** #sqdsq -> http://www.w3.org/2001/XMLSchema#string *()*


**mrNumber** #sqdsq -> http://www.w3.org/2001/XMLSchema#string *()*


**dsq** #sqdsq -> http://www.w3.org/2001/XMLSchema#string *()*


**size** #sqdsq -> http://www.w3.org/2001/XMLSchema#string *()*


**url** #sqdsq -> http://www.w3.org/2001/XMLSchema#string *()*


**name**\_ -> http://www.w3.org/2001/XMLSchema#string *()*


**shortName**\_ -> http://www.w3.org/2001/XMLSchema#string *()*


**dsazdjz** #Part -> http://www.w3.org/2001/XMLSchema#string *()*


**numberOrVolume**(*#sqdsq* | *#zqedzbx*) -> \_ *()*
:   **number** #sqdsq -> http://www.w3.org/2001/XMLSchema#string *()*


    **issue**(*#sqdsq* | *#zqedzbx*) -> http://www.w3.org/2001/XMLSchema#string *()*


    **zsbdgz** #sqdsq -> http://www.w3.org/2001/XMLSchema#nonNegativeInteger *()*

**year** #Date -> http://www.w3.org/2001/XMLSchema#gYear *()*


**month** #Date -> http://www.w3.org/2001/XMLSchema#gMonth *()*


**day** #Date -> http://www.w3.org/2001/XMLSchema#gDay *()*


**city** #Address -> http://www.w3.org/2001/XMLSchema#string *()*


**zdnzadh** #Address -> http://www.w3.org/2001/XMLSchema#string *()*


**country** #Address -> http://www.w3.org/2001/XMLSchema#string *()*


**startPage** #PageRange -> http://www.w3.org/2001/XMLSchema#nonNegativeInteger *()*


**endPage** #PageRange -> http://www.w3.org/2001/XMLSchema#nonNegativeInteger *()*

## Individuals

<rdf:List@ttp://www.w3.org/1999/02/22-rdf-syntax-ns#nil>

---

Generated by OWL2HTML
